# Three-dimensional MoS_2_/Graphene Aerogel as Binder-free Electrode for Li-ion Battery

**DOI:** 10.1186/s11671-019-2916-z

**Published:** 2019-03-08

**Authors:** Yan Zhong, Tielin Shi, Yuanyuan Huang, Siyi Cheng, Chen Chen, Guanglan Liao, Zirong Tang

**Affiliations:** State Key Laboratory of Digital Manufacturing Equipment and Technology, Huazhong University of Science and Technology, Wuhan, 430074 People’s Republic of China

**Keywords:** MoS_2_, Graphene aerogel, Binder-free, Li-ion battery

## Abstract

Hybrid MoS_2_/reduced graphene aerogels with rich micro-pore are fabricated through a hydrothermal method, followed by freeze-drying and annealing treatment. The porous structure could act as an electrode directly, free of binder and conductive agent, which promotes an improved electron transfer, and provides a 3D network for an enhanced ion transport, thus leading to an increased capacity and stable long cycle stability performance. Notably, the specific capacity of MoS_2_/reduced graphene aerogel is 1041 mA h g^−1^ at 100 mA g^−1^. Moreover, reversible capacities of 667 mA h g^−1^ with 58.6% capacity retention are kept after 100 cycles. The outstanding performance is beneficial from the synergistic effect of the MoS_2_ nanostructure and graphene conductive network, as well as the binder-free design. These results provide a route to integrate transition-metal-dichalcogenides with graphene to fabricate composites with rich micro-pores and a three-dimensional network for energy storage devices.

**Electronic supplementary material:**

The online version of this article (10.1186/s11671-019-2916-z) contains supplementary material, which is available to authorized users.

## Introduction

Nowadays, the rapid development of electric vehicles and flexible electronics opens up an opportunity for the development of energy storage devices in the industrial and research communities [1, 2]. Among the various energy storage devices, lithium ion batteries (LIBs) are paid more attention due to their outstanding energy storage capability as well as long cycle life [3–5].

Recently, many researches have focused on high-performance anode materials for LIBs. 2D transition metal dichalcogenides (TMDs), with outstanding electrochemical performance, have won much attention and showed great potential as anode materials for LIBs [6, 7]. Comparing with conventional metal oxides, the metal sulfides with higher conductivity and larger interlayer spacing promote an improved electron transfer and enhanced ion transport [8]. Among the metal sulfides, molybdenum disulfide (MoS_2_) shows great advantages as the anode of LIBs due to its unique layered structure and high capacity (ca. 670 mAh g^−1^). However, its structure is prone to deteriorate during the charge/discharge process due to volume change, leading to a poor cycling stability. Numerous attempts have been conducted to enhance kinetic behaviors of MoS2 as LIBs anode. One method is to synthesis nano-size structure, in order to shorten the diffusion distance of lithium ions [9, 10]. Another method is to incorporate carbon materials to improve the composite conductivity and repress the volume expansion during charge/discharge process [11–13]. Different carbon materials [14–20], including carbon nanotubes [18] and graphene [19, 20], are used to integrate with MoS_2_ and it proves to be in effect. Especially, graphene has drawn much attention benefiting from its outstanding conductivity and high surface area. Recently, graphene has been widely researched in many areas, such as conductive switching [21], photoluminescence [22], chemical cleaning [23], and gas sensing [24] as well as energy storage fields [25]. For instance, Teng et al. prepared MoS_2_ nanosheets on graphene sheets, and a capacity of 1077 mAh g^−1^ at 100 mA g^−1^ after 150 cycles was obtained [26]. Liu et al. fabricated a composite of MoS_2_ and graphene [27], and the reversible capacity of 1300–1400 mAh g^−1^ was obtained. How to incorporate graphene with MoS_2_ to obtain the high-capacity and stable anode material is still an ongoing task [11].

Herein, a facile and low-cost approach is used to prepare a hierarchical nanostructure of MoS_2_/reduced graphene (MoS_2_/RGO) aerogel. With a solvothermal and freezing-drying process, the MoS_2_/RGO aerogel is fabricated and directly acts as the binder-free anode. Such a structure endows the MoS_2_/graphene aerogel with several advantages as an anode material. First, the graphene acts as a matrix to support the MoS_2_ nanostructures, which is beneficial to preventing graphene sheets from restacking. Second, the hierarchical nanostructure provides a good adhesion between graphene and MoS_2_, which ensure a stable structure and thus guarantee a long cycling stability. Third, the graphene with high conductivity promotes an improved electron transfer and acts as a basis to alleviate volume expansion of MoS_2_ in the charge/discharge process. Fourth, such a binder-free design shortens the ion diffusion distance, leading to an enhanced ion transport. The reversible capacity of the as-prepared binder-free MoS_2_/RGO aerogel is up to 667 mA h g^−1^ at 100 mA g^−1^ after 100 cycles. This method provides a route to fabricate the high-performance lithium-ion anode material.

## Materials and Methods

### Synthesis of MoS2/RGO Aerogels

All reagents were of analytical grade. A modified Hummers’ method was used to prepare graphene oxide (GO) for further use [28]. The MoS_2_/RGO aerogels were prepared with a one-step hydrothermal method. In detail, 60 mg of (NH_4_)_2_MoS_4_ were dissolved in 10 mL of *N*, *N*-dimethylformamide (DMF) solvent. Five milliliters of GO aqueous (5 mg mL^−1^) were added, and a homogeneous solution was obtained under sonication for several hours. The solution was put to a Teflon-lined autoclave and sealed. Finally, it was heated in the oven at 200°C for 12 h. MoS_2_/RGO hydrogels were obtained through washing with ethanol and D.I. water. Through freeze-drying and annealing in 700°C for 2 h, the final MoS_2_/RGO aerogels were obtained. As a comparison, the MoS_2_ powder was prepared with the same steps except adding GO.

### Characterization

A thin piece of MoS_2_/RGO film which was cut from the MoS2/RGO aerogels was used to carry out further characterization. Field mission scanning electron microscopy (FESEM, JEOL JSM-6700F) and field-emission transmission electron microscopy (FETEM, FEI, Tecnai G2 F30) were used to characterize the obtained samples. XRD analysis (PANalytical PW3040/60) with Cu Kα radiation (*λ* = 1.5406 Å) from 10° to 80° was used to confirm the substance of the MoS2/RGO film and MoS2 powder.

### Electrochemical Measurements

The MoS2/RGO film was directly used as a binder-free anode, without any binder and conductive agent. It was assembled into a coin-type half-cell in a glove box, with a lithium foil acting as counter electrode and Celgard 2400 polymer as separator. The electrolyte consisted of 1 M LiPF6 in ethylene carbonate (EC) and diethyl carbonate (DEC). After assembly, the cell was aged 24 h in the glove box for further measurements. The galvanostatic charge/discharge (GCD) measurements were carried out with a battery measurement system (Land, China), and cyclic voltammetry (CV) testings were conducted with Autolab workstation (PGSTAT-302N). The testing was conducted in the potential range of 0.01–3.0 V (vs Li1/Li). Electrochemical impedance spectra (EIS) experiments were carried out with 10 mV amplitude in the frequency from 100 kHz to 0.01 Hz.

## Results and Discussion

**Fig. 1:**
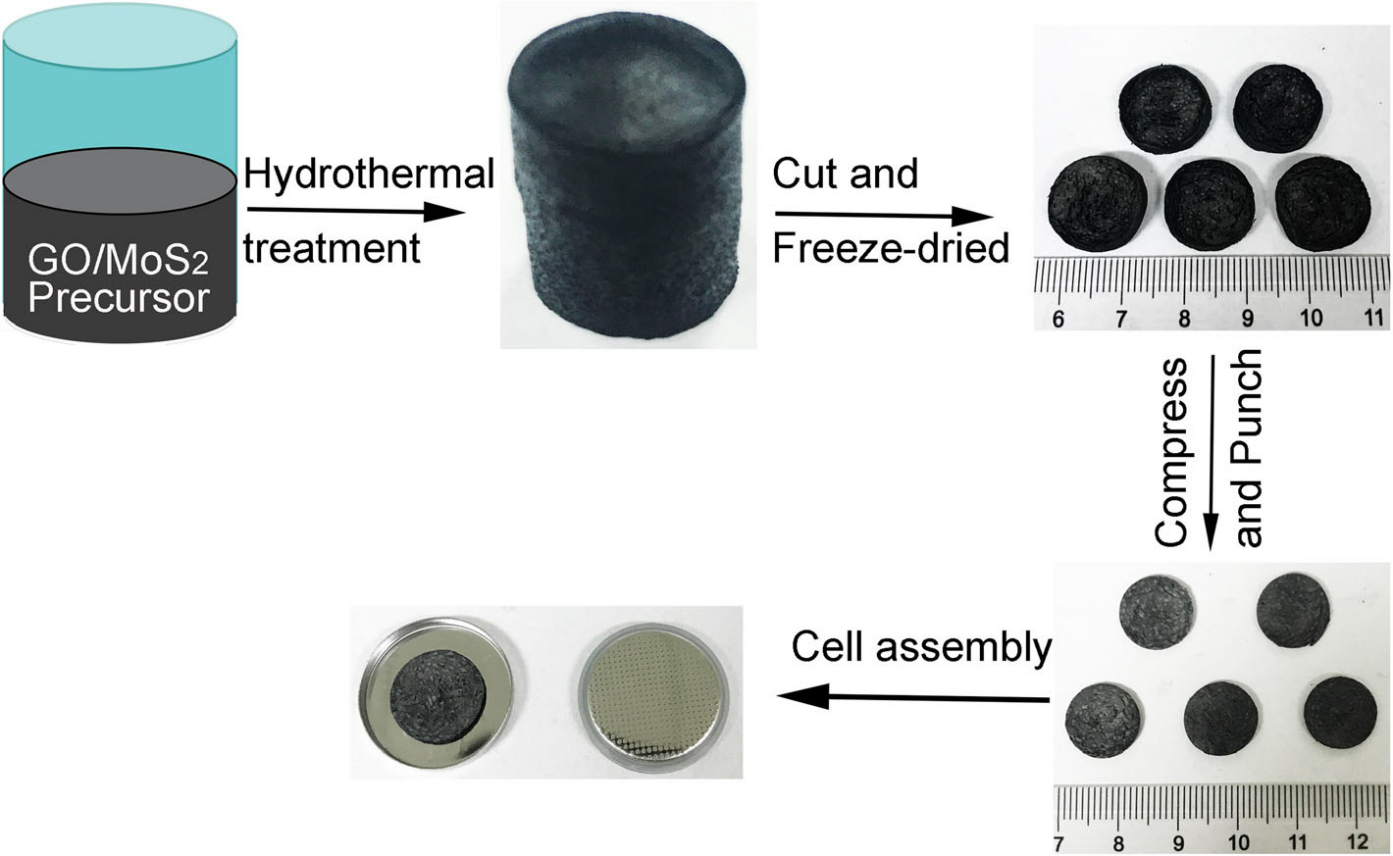
Schematic of fabrication of hybrid nanostructure of MoS2/RGO

The MoS2/RGO aerogels were fabricated with a hydrothermal method, freeze-drying and heat treatment. Figure 1 displayed the preparation process of the MoS2/RGO electrode. Detailed methods were described on the Materials and methods. As shown in Additional file 1: Figure S1 and Additional file 2: Figure S2, the obtained MoS_2_/RGO aerogel could keep integrate structure. The excellent mechanical behavior was beneficial from the rich porosity of the whole structure and the interconnection of graphene layers, showing great potential as a binder-free electrode.

**Fig. 2:**
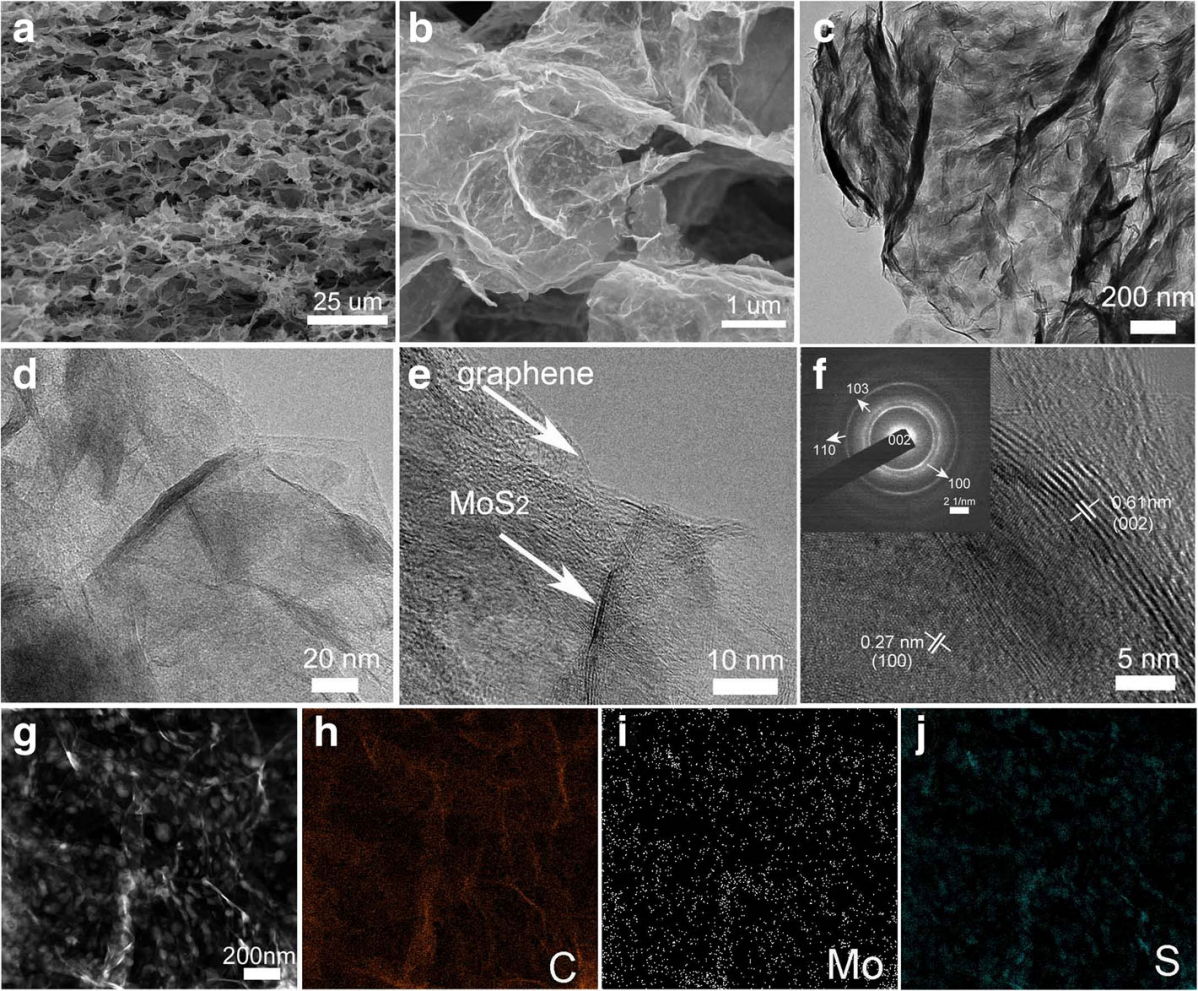
**a**, **b** SEM images and **c**, **d**, **e**, **f** TEM and HRTEM images of the MoS2/RGO sample. **g**–**j** TEM-EDX mapping of Mo, S, and C elements. The inset in **f** is the corresponding SAED pattern

Figure 2 presented the morphology of MoS_2_/rGO aerogel. A porous structure with wrinkled graphene layers interconnected with each other was observed (Fig. 2a), where MoS_2_ nanostructures covered the whole graphene layers. The microstructure of MoS_2_/RGO aerogels was further confirmed with TEM (Additional file 3: Figure S3). As displayed in Fig. 2c and d, the MoS_2_ nanostructures were distributed on the graphene even after long-time ultrasonication, illustrating the strong interaction of MoS_2_ on graphene. The high-resolution TEM image was displayed in Fig. 2f. The graphene layers were covered with MoS_2_ nanostructures, where lattice spacings of 0.61 and 0.27 nm were observed, which were responsible for (002) and (100) planes of MoS_2_ [29]. The SAED pattern (inset of Fig. 2f) presented several diffraction rings, which was well corresponding to MoS_2_ planes [30]. These results illustrated that MoS2 nanostructures on graphene layer exhibited a good crystallinity. The elemental distribution of the aerogel was detected (Fig. 2g–j) where Mo, S, and C elements were almost overlapped with the whole structure, suggesting the successful fabrication of the composite.

**Fig. 3:**
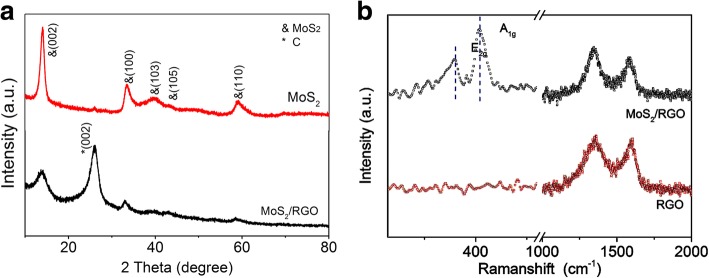
**a** XRD patterns of MoS2/RGO and MoS2 samples. **b** Raman spectra of the MoS2/RGO and MoS2

X-ray diffraction (XRD) experiments were also carried out. As shown in Fig. 3a, the XRD patterns of the MoS_2_ powder could be responsible for hexagonal 2H–MoS_2_ (JCPDS 37-1492). The strong reflection peak at 2*θ* = 14.2^o^ belonged to the (002) plane, with a d-spacing of 0.62 nm. MoS_2_/RGO composite showed the similar crystalline structure of pure MoS_2_, indicating a layered structure. Comparing with the MoS_2_ samples, an obvious peak in 26.3° was observed in the MoS2/RGO samples, which could be the (002) diffraction peak of graphene, revealing the graphene substance in the composites [31]. It was worth pointing out that the obvious peak at 14.4°, 32.7° and 58.3° were ascribed to the (002), (100) and (110) diffraction peak of MoS_2_, which was consistent with the previous SAED pattern results. Notably, the MoS_2_ (002) reflection peak, which indicated a stacked nature of layered MoS2, was weakened for the MoS_2_/RGO composite, suggesting the formation of a few-layer MoS2 structure [26, 32]. The peaks of graphene were more obvious than the MoS_2_, further confirming that the MoS_2_ was wrapped by graphene layer in the MoS_2_/RGO aerogels [26, 32].

To further confirm the nature of MoS_2_ nanostructure and graphene layer, Raman spectroscopy measurements were also carried out [33–35]. As shown in Fig. 3b, the MoS_2_/RGO aerogel showed the E_2g_ and A_1g_ peaks of MoS_2_ at the frequencies of 380.2 and 403.6 cm^−1^ [18, 36]. Notably, it had been reported that the single-layer MoS_2_ nanostructure with different fabrication method would display an A_1g_ peak at 402–404 cm^−1^ [37–39], further identifying the few layer of MoS_2_ crystals in the MoS_2_/RGO aerogel. Besides, the peaks at 1354.3 cm^−1^ and 1591.6 cm^−1^ were observed in Fig. 3b, which were characteristic peaks of the D- and G-bands of graphene [40–42]. The intensity ratio *I*_D_/*I*_G_ was usually associated with the graphene defects [35]. The value was calculated to be 1.08, indicating the reduced graphene with some defects [34].

**Fig. 4:**
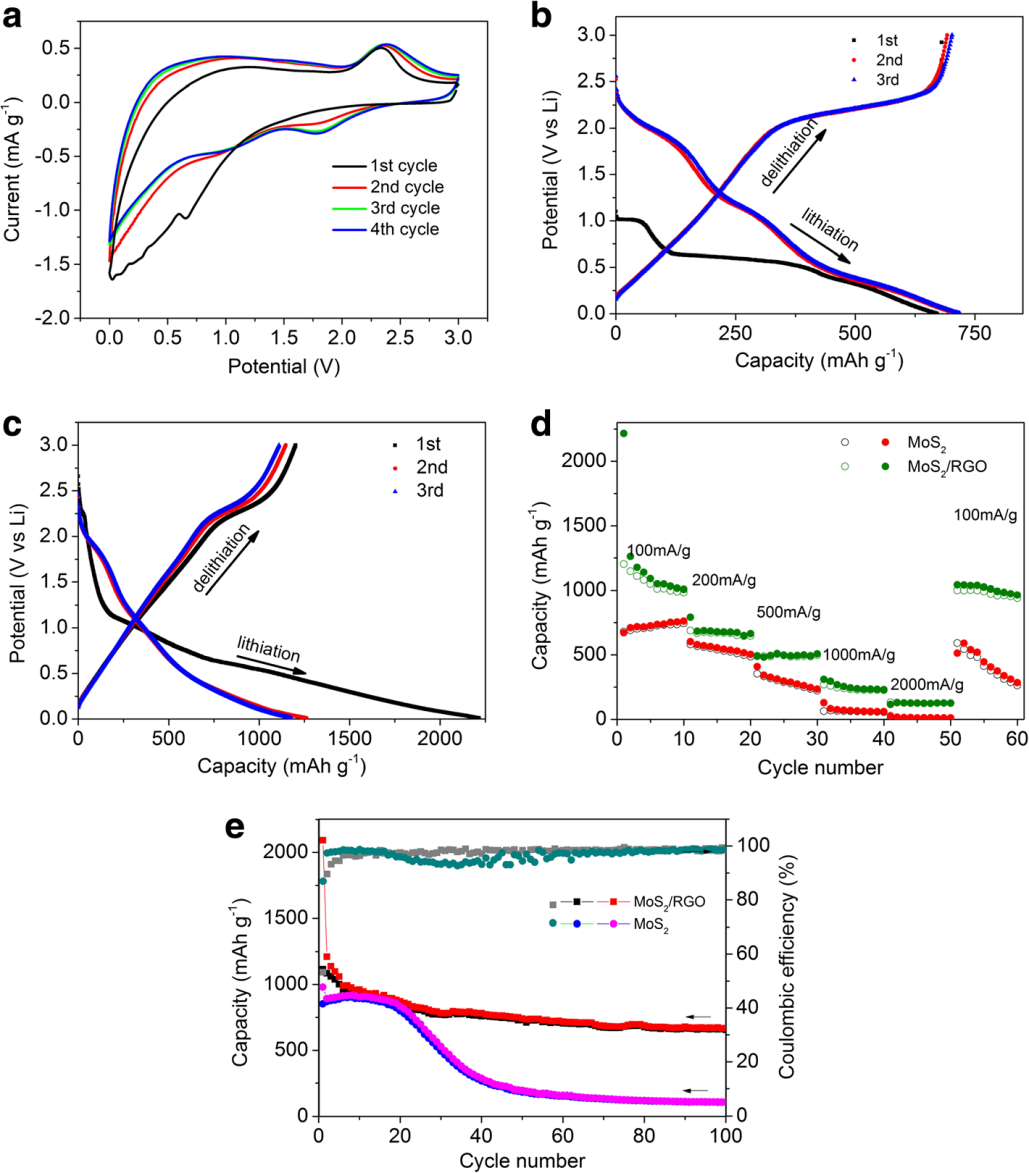
The first three cyclic voltammograms of MoS2/RGO aerogel at a scan rate of 0.5 mV s-1 (**a**). Galvanostatic charge and discharge curves of MoS2/RGO aerogel (**b**) and MoS2 (**c**) electrodes at a current density of 100 mA g-1. **d** Rate performances of MoS2/RGO aerogel and MoS2 electrodes at different current densities. **e** Cycling performance of MoS2/RGO aerogel and MoS2 electrodes at a constant current density of 100 mA g-1

To demonstrate the performance of MoS2/RGO electrode, CV measurements at a scan rate of 0.5 mV s^−1^ were carried out. Figure 4a showed the first three CV curves of MoS2/RGO composite. A broad shoulder peak was observed at 0.95 V when there were reduction peaks at 0.65 V in the first cathodic sweep of the MoS_2_/RGO electrode. The peak at 0.95 V was related with Li+ intercalation into MoS_2_ interlayer space to form LixMoS_2_, with a phase transformation process to become 1T(octahedral) structure of LixMoS2 from 2H (trigonal prismatic) [43, 44]. The other peak at 0.65 V was accompanied with the process to form Li_2_S and metallic Mo from LixMoS_2_ [45–47]. In the following discharge scans, there were reduction peaks located at 1.80 V and 1.05 V, indicating a different reaction process. One pronounced peak at 2.34 V was observed for the MoS_2_/RGO electrode in the reverse anodic scans, indicating the formation of sulfur [43]. It could be inferred that sulfur, Mo, and few MoS_2_ were formed after the first cycle and they were kept the same in subsequent cycles [36, 48–50]. In addition, the discharge curves were identical except for the first one, indicating the electrochemical stability for the MoS_2_/RGO composite. The first three GCD curves of the MoS2/RGO and MoS2 electrodes were shown in Fig. 4b and c. In the first discharge cycle of the MoS2 electrode, two potential plateaus were observed at 1.05 V and 0.65 V (Fig. 4b). The 1.05 V plateau was accompanied with the process of forming LixMoS_2_, and the plateau at 0.65 V was related with the reaction of forming Mo particles from MoS_2_. A slope potential curve was observed below 0.52 V in the first discharge cycles, meaning the appearance of gel-like polymeric layer due to the degradation of electrolyte [51–53]. The MoS_2_ electrode showed plateaus at 2.0, 1.20 and 0.45 V in the following discharge curves. In the charge process, an obvious plateau at 2.35 V was observed for the MoS2 electrode. For the MoS2/RGO electrode (Fig. 4c), there was no obvious potential plateau during the first discharge cycle, except for a week plateau at 1.1–0.6 V, which was mainly ascribed to the overlapping lithium process in MoS2 and RGO [54]. MoS2/RGO electrode displayed a plateau at 1.95 V in the following discharge cycles, in agreement with the CV results. During the charge cycles, the MoS_2_/RGO electrode showed a plateau at 2.2 V. Figure 4c showed the discharge and charge capacity of MoS_2_/RGO and MoS_2_ electrode. MoS_2_/RGO electrode delivered 2215 mAh g^−1^ discharge capacity in the first discharge cycle, with a reversible charge capacity of 1202 mAh g^−1^. The corresponding values for the MoS_2_ were 671.1 mAh g^−1^ and 680.5 mAh g^−1^, respectively. The irreversible processes in the first cycle, such as decomposition of electrolyte and the formation of SEI film, lead to irreversibility [55, 56].

The rate performances of MoS_2_/RGO electrode and MoS_2_ electrodes were shown in Fig. 4d. Comparing with single MoS_2_ electrodes, MoS2/RGO electrodes delivered higher capacities. A capacity of 1041 mAh g^−1^ at 100 mA g^−1^ was kept after 50 discharge/charge cycles for the MoS_2_/RGO electrode, indicating a good electrochemical reversibility as well as a long cycle stability. By comparison, the MoS_2_ electrode only kept 512 mAh g^−1^ capacity at 100 mA g^−1^ after 50 cycles. Moreover, the specific capacity of the MoS2 electrode decreased a lot when the current decreased from 2000 mA g^−1^ to100 mA g^−1^. The cycling results conducted at 100 mA g^−1^ were shown in Fig. 4e. The MoS_2_ electrode showed a poor cycling performance. There was nearly no decrease in its initial 20 cycles. However, the reversible (charge) capacity decreased from 892 mAh g^−1^ to 110 mAh g^−1^ after 100 cycles, with only 12.3% capacity retention. On the contrary, the MoS_2_/RGO electrodes displayed an improved cyclic stability. A reversible capacity of 667 mAh g^−1^, with a 58.6% capacity retention was obtained after 100 cycles. The rate performances and cycling stability of pure RGO electrode were also displayed in Additional file 4: Figure S4. The RGO electrode delivered a reversible charge capacity of 297.8 mAh g^−1^ at 100 mA g^−1^. When the current density reversed from 2000 mA g^−1^ to100 mA g^−1^, the specific capacity of 202.2 mAh g^−1^ was kept for the RGO electrode. Table 1 showed a comparison of the capacity performance about the binder-free MoS2/RGO and other materials based on MoS2/rGO listed in the literature [57–63]. It could be seen that the binder-free MoS2/RGO electrode showed high capacity compared with other porous MoS2/RGO composites ever reported. These results illustrated the successful introduction of RGO, and the important role it played in the delithium-lithium process [57]. Firstly, the graphene layer with highly porous architecture provided rich active sites for the MoS_2_ nanostructure, which was beneficial to preventing aggregation of MoS_2_. Secondly, the graphene with good conductivity reduced transfer resistance and promoted electron transmission and ion transport, leading to an improved rate capability. Thirdly, the RGO aerogel with multi-scale porous structure acted as an elastic buffer layer, which effectively restrained the volume expansion during the delithium-lithium process, and thus lead a better cycling stability.

**Table 1:** Comparison of the capacity of MoS2-graphene composites materials for Li-ion Battery

Material	Method	Current density	Capacity	Reference
MoS2/Graphene heterostructure	Hydrothermal	100 mA g^–1^	786 mAh g^–1^	1 [58]
MoS2-rGO composites	Microwave annealing	100 mA g^–1^	908 mA h g^–1^	2 [59]
MoS2-RGO composites	Supercritical methanol route	50 mA g^–1^	896 mA h g^–1^	3 [60]
Layer-by-layer MoS2/rGO hybrids	Intercalation exfoliation	100 mA g^–1^	940 mA h g^–1^	4 [61]
MoS2-graphene hybrids	High temperature heat-treatment	100 mA g^–1^	800 mAh g^–1^	5 [62]
MoS2-graphene hybrid nanosheets	Hydrothermal	100 mA g^–1^	902 mA h g^–1^	6 [63]
Binder-free MoS2/rGO hybrids	Hydrothermal	100 mA g^−1^	1041 mAh g^−1^	This work

**Fig. 5:**
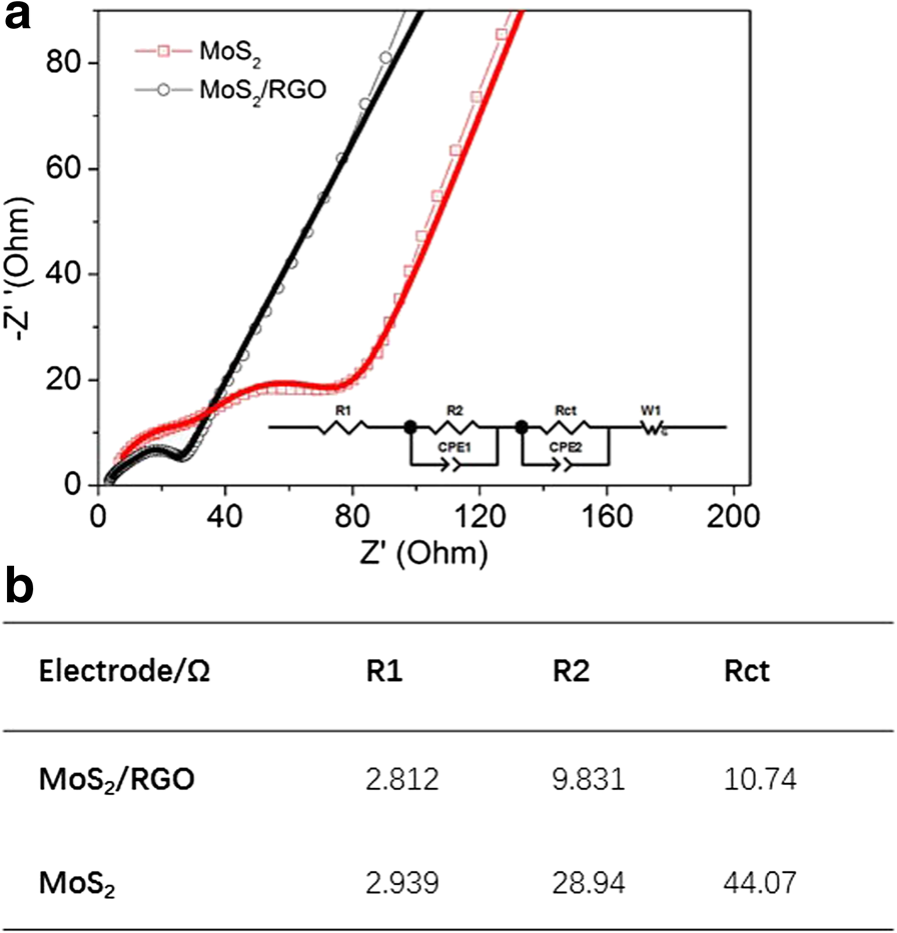
**a** Nyquist plots of MoS2/RGO and MoS2 electrodes at fully charged state after 100 cycles at 100 mA g^−1^, and **b** values of R1, R2, and Rct obtained by fitting data according to the equivalent circuit model presented in **a**

Electrochemical impedance spectra (EIS) measurements were also conducted for the samples. Figure 5a showed the Nyquist plots of MoS_2_/RGO and MoS_2_ electrodes after 100 discharge-charge cycles at 100 mA g^−1^. The first semicircle represented lithium ion migration resistance through the SEI films (R1), while the second semicircle stood for the resistance of charge transport (Rct). R2 was related with the resistance of electrolyte [26]. ZView software was used to fit the curves of MoS_2_/RGO and MoS_2_ electrodes. The fitted values were listed in the Fig. 5b. From the table, the Rct of the MoS_2_/RGO electrode (10.74Ω) was smaller than MoS_2_ (44.07 Ω), indicating that rGO could bring an improved charge transfer process during discharge-charge actions and thus show a good rate capability.

To investigate the impact of repeated charge/discharge processes on the as-prepared samples, FESEM were conducted on the samples after 100 cycles at 100 mA g^−1^ (Additional file 1: Figure S1). MoS2/ RGO electrode kept a well structure without any cracks. The cross-sectional FESEM pictures in Additional file 1: Figure S1c and d showed the high-compressible graphene layer where nanoparticles were distributed. On the contrary, severe cracks were observed on the pristine MoS_2_ electrode in Additional file 1: Figure S1e and f. It was mainly because the volume expansion of active material during cycling, thus leading to particles aggregation. The above results illustrated the important role of graphene layer in inhibiting the volume expansion in the cycling process (Additional file 5: Figure S5).

## Conclusion

In summary, hybrid MoS_2_/RGO aerogels with rich micropores have been fabricated. The prepared aerogels are used as electrodes without any binder and conducting agent. Such a nanostructure design with abundant micro-pores is not only beneficial to providing 3D network for enhanced electron transfer, but also can shorten the transport distance, thus leading to an improved electrochemical rate and stable performance as the anode electrodes for LIBs. MoS_2_/RGO aerogel delivers specific capacities of 1041 mA h g^−1^ at 100 mA g^−1^, which is ascribed to the synergistic effect of MoS2 nanostructure and conductive graphene, as well as the binder-free design with abundant micro-pores. The study offers useful insights for realizing high-performance anode electrodes for LIBs with high capacity and long cycle stability.

## Additional files

Additional file 1: **Figure S1.** Mechanical performance of the MoS2/RGO aerogel under the finger compression. (JPG 70 kb)

Additional file 2: **Figure S2.** Mechanical performance of the MoS2/RGO aerogel before compression (a), under compression (b), and after compression (c). (PNG 2203 kb)

Additional file 3: **Figure S3.** (a) TEM picture of the MoS_2_/RGO sample. (b) TEM-EDS mapping of Mo, S, C elements. (c) EDX spectra of the MoS_2_/RGO sample. (JPG 304 kb)

Additional file 4: **Figure S4.** (a) Rate performances of RGO aerogel electrodes at different current densities. (b) Cycling performance of RGO electrodes at a constant current density of 100 mA g^−1^. (JPG 114 kb)

Additional file 5: **Figure S5.** FESEM images of (a, b) MoS_2_/RGO electrode, (c, d) cross-sectional images of MoS_2_/RGO and SEM images of (e, f) bare MoS_2_ electrode after 100 cycles performed with a current density of 100 mA g^−1^. (JPG 1649 kb)

## Abbreviations

2H: Trigonal prismatic
CV: Cyclic voltammograms
EIS: Electrochemical impedance spectroscopy
GCD: Galvanostatic charge/discharge
GO: Graphene oxide
HRTEM: High-resolution TEM
LIBs: Lithium ion batteries
MoS_2_: Molybdenum disulfide
MoS_2_/RGO: MoS_2_/ reduced graphene
R1: Lithium ion migration resistance through the SEI films
R2: The resistance of electrolyte
Rct: The resistance of charge transport
SAED: Selected area electron diffraction
TMDs: 2D transition metal dichalcogenides
XRD: X-ray diffraction
