# Supplementary figures and images for: Three-dimensional MoS2/Graphene Aerogel as Binder-free Electrode for Li-ion Battery
Source: Nanoscale Res Lett. 2019 Mar 8;14:85. doi: 10.1186/s11671-019-2916-z (PMC6408559; doi:10.1186/s11671-019-2916-z)

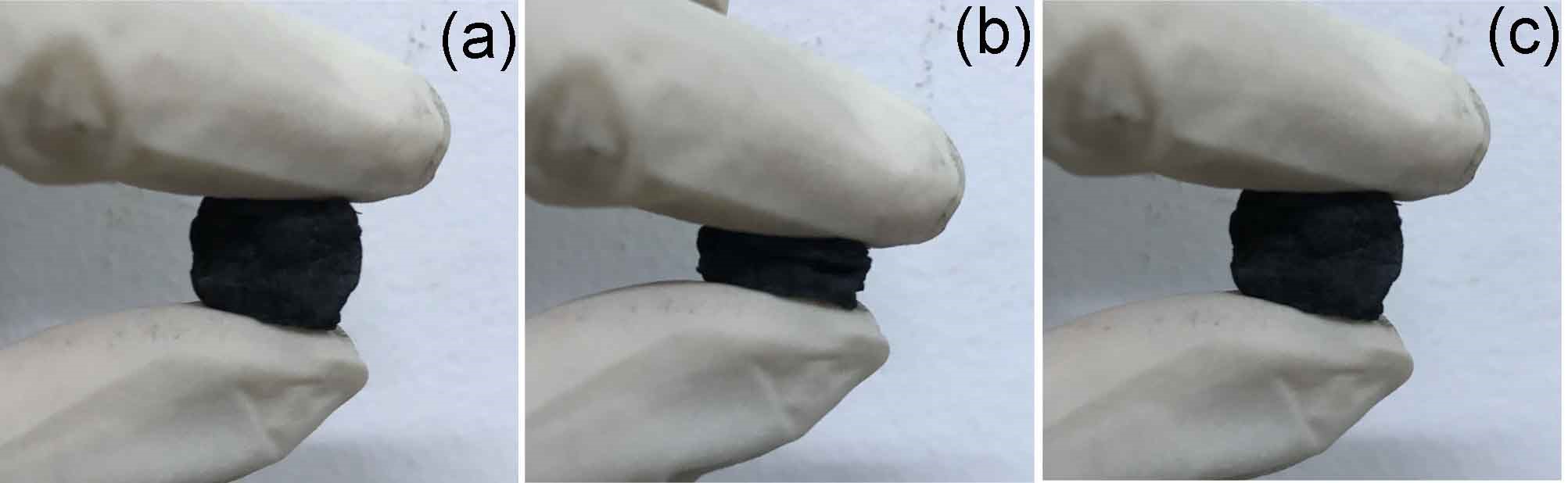

Supplement: Supplementary file 1 — Figure S1. Mechanical performance of the MoS2/RGO aerogel under the finger compression. (JPG 70 kb) [file 11671_2019_2916_MOESM1_ESM.jpg]

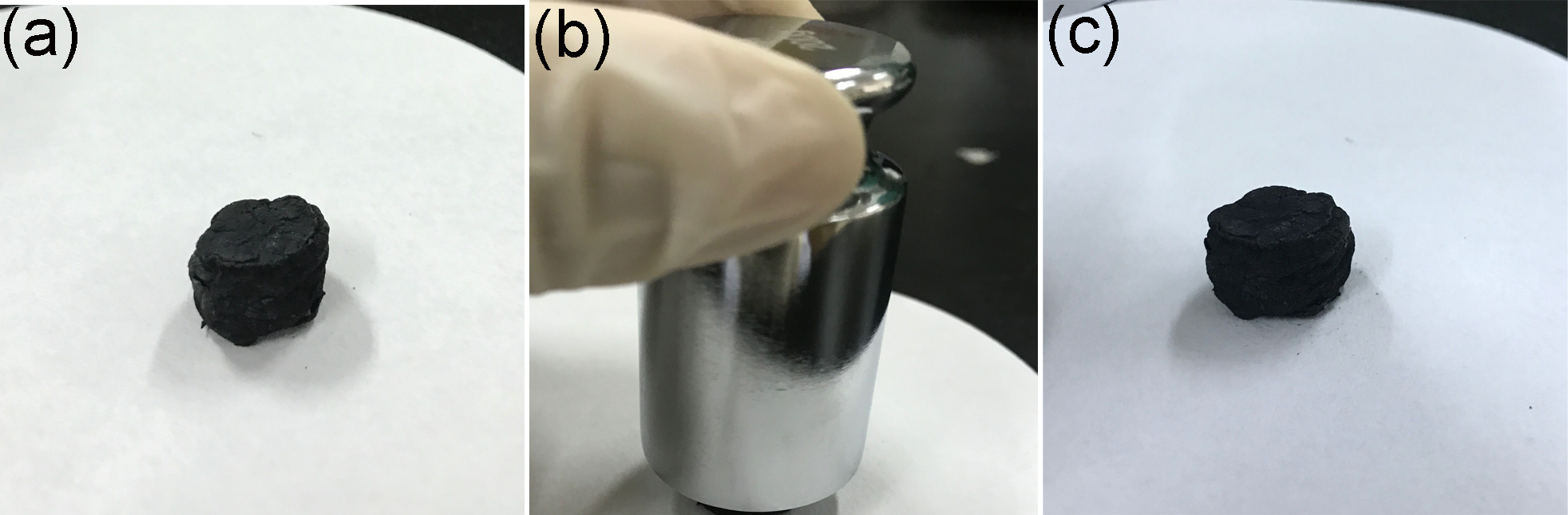

Supplement: Supplementary file 2 — Figure S2. Mechanical performance of the MoS2/RGO aerogel before compression (a), under compression (b), and after compression (c). (PNG 2203 kb) [file 11671_2019_2916_MOESM2_ESM.png]

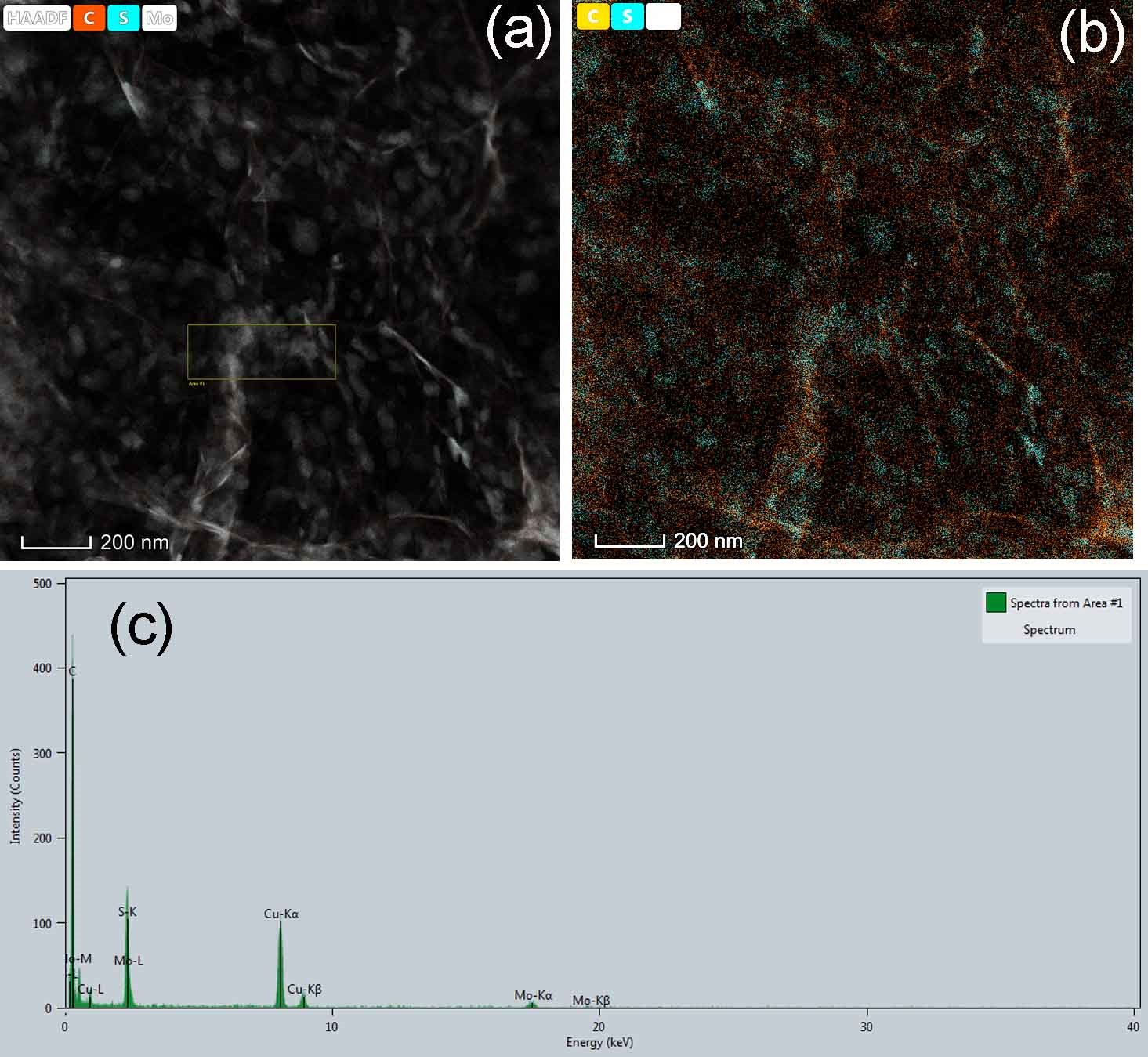

Supplement: Supplementary file 3 — Figure S3. (a) TEM picture of the MoS2/RGO sample. (b) TEM-EDS mapping of Mo, S, C elements. (c) EDX spectra of the MoS2/RGO sample. (JPG 304 kb) [file 11671_2019_2916_MOESM3_ESM.jpg]

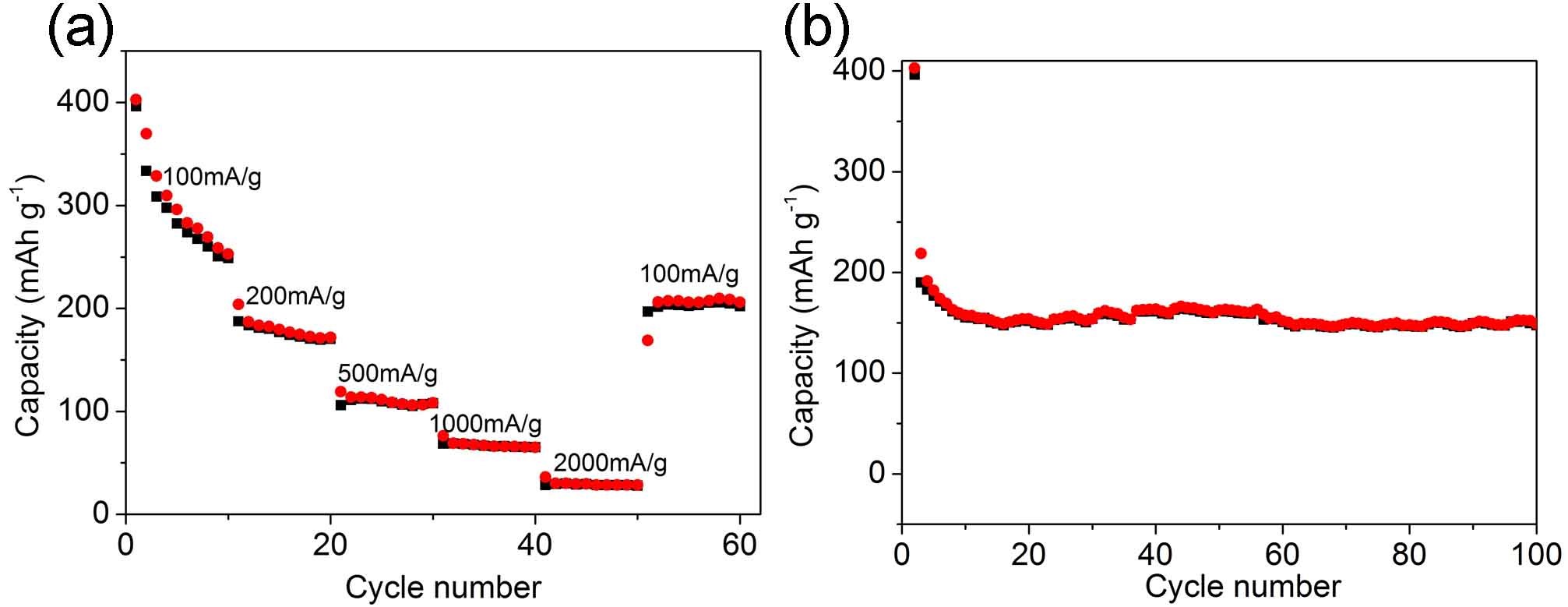

Supplement: Supplementary file 4 — Figure S4. (a) Rate performances of RGO aerogel electrodes at different current densities. (b) Cycling performance of RGO electrodes at a constant current density of 100 mA g−1. (JPG 114 kb) [file 11671_2019_2916_MOESM4_ESM.jpg]

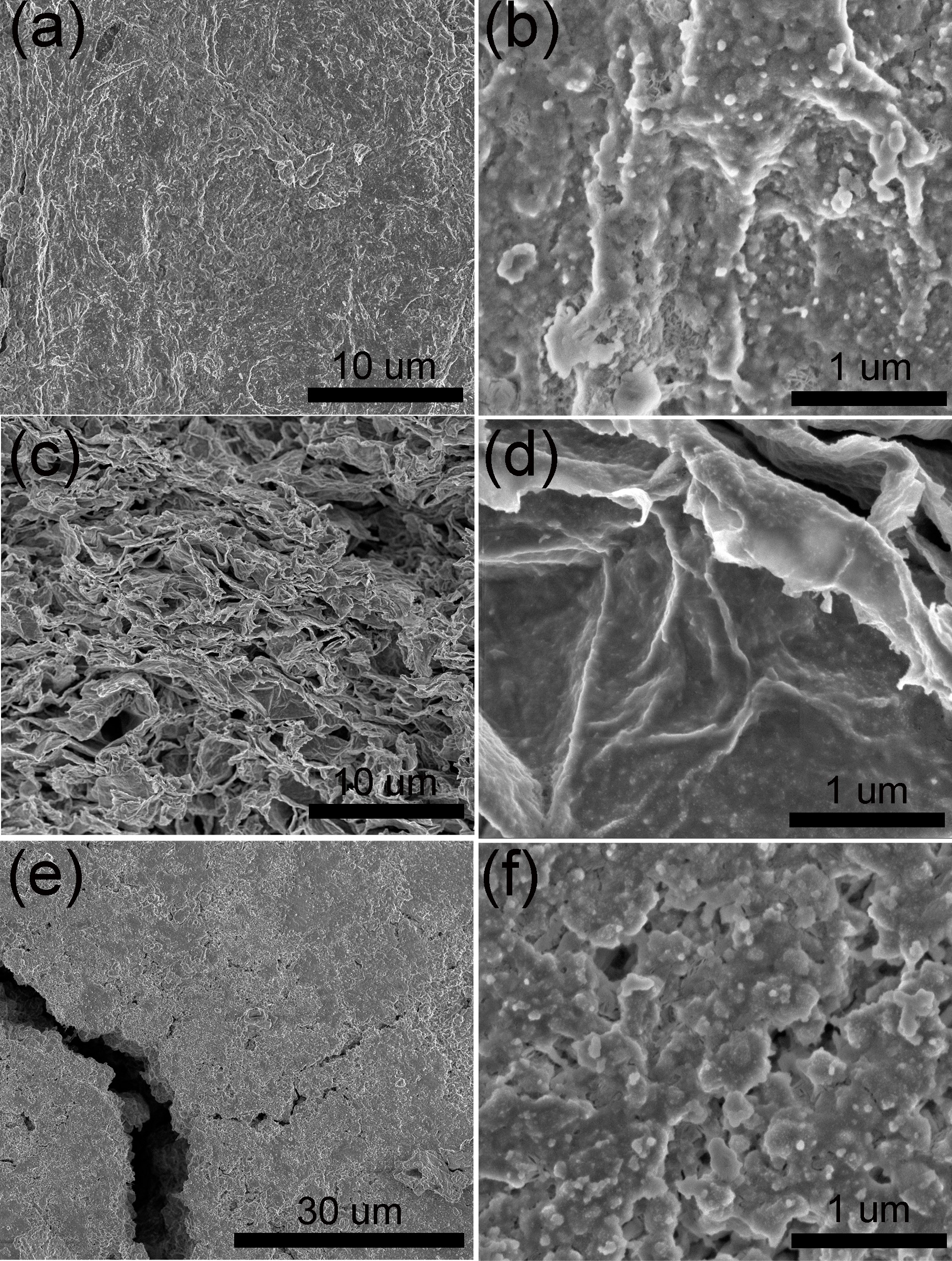

Supplement: Supplementary file 5 — Figure S5. FESEM images of (a, b) MoS2/RGO electrode, (c, d) cross-sectional images of MoS2/RGO and SEM images of (e, f) bare MoS2 electrode after 100 cycles performed with a current density of 100 mA g−1. (JPG 1649 kb) [file 11671_2019_2916_MOESM5_ESM.jpg]
